# Supplementary material for: ALBI Score Is a Strong Predictor of Toxicity Following SIRT for Hepatocellular Carcinoma
Source: Cancers (Basel). 2021 Jul 28;13(15):3794. doi: 10.3390/cancers13153794 (PMC8345032; doi:10.3390/cancers13153794)

cancers

MDPI

Article

ALBI Score Is A Strong Predictor of Toxicity Following SIRT for Hepatocellular Carcinoma

Céline Lescure [1], Florian Estrade [1], Maud Pedrono [1], Boris Campillo-Gimenez [2], Samuel Le Sourd [1], Marc Pracht [1], Xavier Palard [3], Héloïse Bourien [1], Léa Muzellec [1], Thomas Uguen [4], Yan Rolland [5], Etienne Garin [3] and Julien Edeline [1,*]

Supplementary materials

Table S1. Scoring system used for C-P in this study

	1 point	2 points	3 points
Albumin	> 35 g/L	35–28 g/L	< 28 g/L
Bilirubin	< 34 mcmol/L	34–51 mcmol/L	> 51 mcmol/L
Coagulation:<br>-INR<br>-Prothrombin Time, as a percentage relative to control	< 1.7<br>> 50%	1.7–2.3<br>40–50%	> 2.3<br>< 40%
Ascites	None	Medically controlled	Refractory
Encephalopathy	None	Grade 1 or 2 (or medically controlled)	Grade 3 or 4 (or refractory)

Table S2. Causes of death.

**Cause of death**	**ALBI Grade 1**<br>**n=88**	**ALBI Grade 2**<br>**n=130**	**ALBI Grade 3**<br>**n=4**
Liver failure due to Selective internal radiation therapy		2	
Liver failure due to cancer progression		3	
Liver failure (indeterminate cause)		2	
Esophageal varices bleeding	1		
Stroke		1	
Indeterminate cause		2	

**Cause of death**	**C-P A5**<br>**n=131**	**C-P A6**<br>**n=70**	**C-P B**<br>**n=21**	**C-P C**<br>**n=0**
Liver failure due to SIRT		2		
Liver failure due to cancer progression	1	2		
Liver failure (indeterminate cause)	1	1		
Esophageal varices bleeding	1			
Stroke		1		
Indeterminate cause	2			

Figure S1. – Flow-chart of the study.

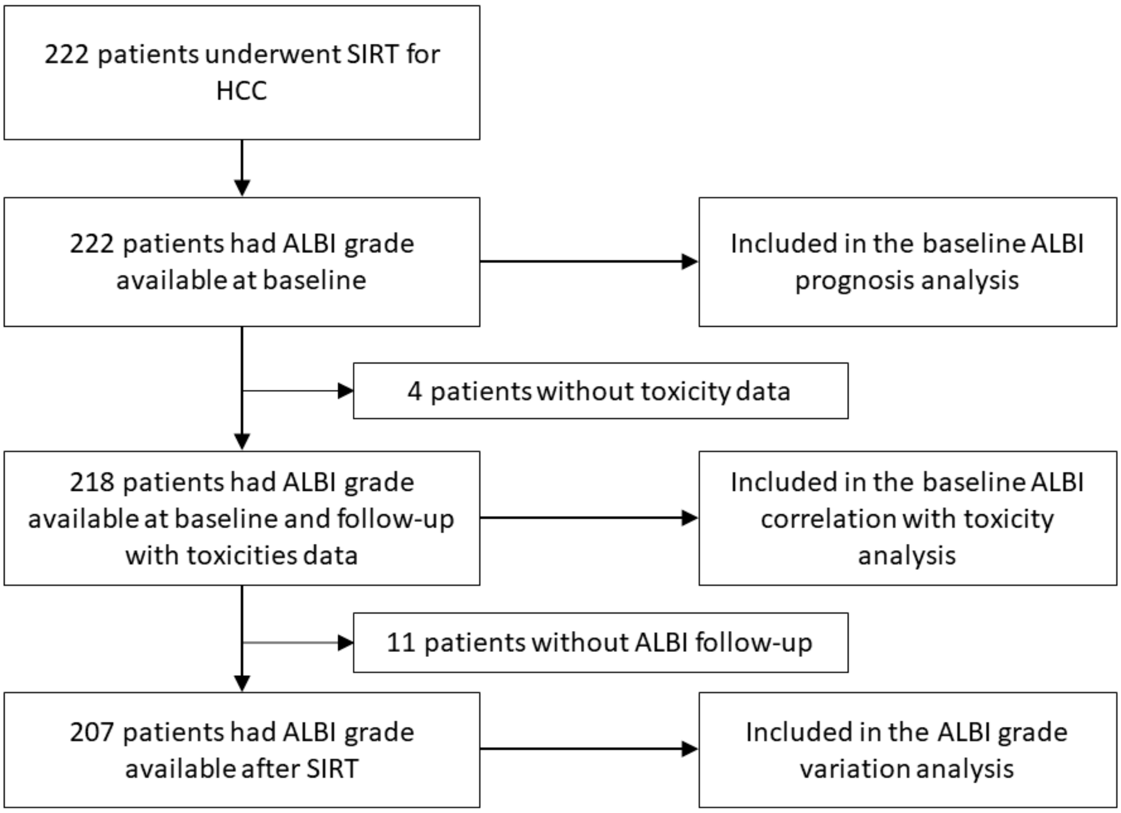

Supplement: Supplementary file 1 [file cancers-13-03794-s001.zip › cancers-1321820-supplementary.pdf]
